# It’s a match! Simulating compatibility-based learning in a network of networks

**DOI:** 10.1007/s00191-018-0579-z

**Published:** 2018-06-14

**Authors:** Michael P. Schlaile, Johannes Zeman, Matthias Mueller

**Affiliations:** 1 Institute of Economics (520i) and Institute of Economic and Business Education (560 D), University of Hohenheim, Wollgrasweg 23, 70593 Stuttgart, Germany; 2 Institute for Computational Physics, University of Stuttgart, Allmandring 3, 70569 Stuttgart, Germany; 3 Institute of Economics (520i), University of Hohenheim, Wollgrasweg 23, 70593 Stuttgart, Germany

**Keywords:** Agent-based modeling, Cognitive distance, Exploitation, Exploration, Innovation, Innovation networks, Knowledge compatibility, Knowledge diffusion, Knowledge networks, Learning, Memetics, Network-of-networks, C63, D83, D85, L14, O33

## Abstract

In this article, we develop a new way to capture knowledge diffusion and assimilation in innovation networks by means of an agent-based simulation model. The model incorporates three essential characteristics of knowledge that have not been covered entirely by previous diffusion models: the network character of knowledge, compatibility of new knowledge with already existing knowledge, and the fact that transmission of knowledge requires some form of attention. We employ a network-of- networks approach, where agents are located within an innovation network and each agent itself contains another network composed of knowledge units (KUs). Since social learning is a path-dependent process, in our model, KUs are exchanged among agents and integrated into their respective knowledge networks depending on the received KUs’ compatibility with the currently focused ones. Thereby, we are also able to endogenize attributes such as absorptive capacity that have been treated as an exogenous parameter in some of the previous diffusion models. We use our model to simulate and analyze various scenarios, including cases for different degrees of knowledge diversity and cognitive distance among agents as well as knowledge exploitation vs. exploration strategies. Here, the model is able to distinguish between two levels of knowledge diversity: heterogeneity within and between agents. Additionally, our simulation results give fresh impetus to debates about the interplay of innovation network structure and knowledge diffusion. In summary, our article proposes a novel way of modeling knowledge diffusion, thereby contributing to an advancement of the economics of innovation and knowledge.

## Introduction

Knowledge is important for the economic system both as input and output (see, e.g., Ancori et al. 2000; Antonelli and Link 2015; Barley et al. 2017; Foray 2004; Mokyr 2002, 2017; Smith 2000) and a central building block of innovation and economic evolution (e.g., Audretsch and Feldman 1996; Dosi 1988; Jensen et al. 2007; Lundvall 2004, 2016; Lundvall and Johnson 1994). Consequently, many authors study knowledge dynamics as a fundamental pillar of innovation both from empirical and theoretical perspectives (see, e.g., Morone and Taylor 2010, for a review). Recently, several agent-based models and simulations have emerged that aim to capture various dynamics of knowledge creation and diffusion in (innovation) networks (e.g., Ahrweiler et al. 2016; Bogner et al. 2018; Cowan and Jonard 2004; Gilbert et al. 2007, 2014; Luo et al. 2015; Morone and Taylor 2004, 2010; Mueller et al. 2017; Schmid 2015; Tur and Azagra-Caro 2018; Vermeulen and Pyka 2017). Knowledge and information as well as their diffusion can be modeled in various forms (see, e.g., Cowan and Jonard 2004; Ferrari et al. 2009; Morone et al. 2007; Weng 2014, for different approaches). In previous models, knowledge has often been represented as a vector of knowledge types or categories (e.g., Cowan and Jonard 2004; Luo et al. 2015; Mueller et al. 2017). However, as Piergiuseppe Morone and Richard Taylor (2010, p. 37) note: “considering knowledge as a number (or a vector of numbers) ... restricts our understanding of the complex structure of knowledge generation and diffusion”. Arguably, when representing knowledge itself as an easily quantifiable cumulative entity in terms of numbers or vectors, the analysis of knowledge diffusion processes may provide an incomplete picture. There are many examples where knowledge generation and diffusion involves more than “stockpiling” additional pieces of knowledge, for example, by establishing meaningful connections and complex relations (cf. *relational knowledge*, Halford et al. 2010). Consequently, knowledge is not only cumulative (e.g., Boschma 2005; Foray and Mairesse 2002), it can also be tacit or sticky (see, e.g., Antonelli 2006; Cowan et al. 2000; Polanyi 1966; Szulanski 2003; von Hippel 1994), and learning must be considered as a path-dependent process (e.g., Baddeley 2010, 2013; Boschma and Lambooy 1999; Dosi et al. 2001; Rizzello 2004; Hayek 1952).

The aim of our article is to contribute to this broad line of research by proposing a *meso-analytic*^1^ agent-based simulation model that presents an alternative approach to modeling knowledge and its diffusion by taking into account the relational, cumulative, and path-dependent aspects of (social) learning. More specifically, we explicitly take into account the following inherent characteristics of knowledge:

- The network character of knowledge,
- compatibility of newly acquired with already existing knowledge (an important aspect of path dependence in social learning processes),
- and competition among knowledge units for attention.

Consequently, the central purpose of our article is to shed light on the potential implications and advantages of modeling knowledge and its diffusion differently. We develop a model that can more adequately capture the complexity of diffusion processes resulting from incorporating these three characteristics of knowledge. At the same time, the model is kept simple enough to be analyzed in a conclusive manner.

The article is structured as follows: Section 2 presents the theoretical background of our approach. In particular, we review important literature and elucidate the article’s research focus (Section 2.1), motivation and foundations (Section 2.2), and then describe our model (Section 2.3). Subsequently, in Section 3, we present the analyses of the simulation results. More precisely, in Section 3.1, we start with the results of a baseline scenario that aims to illuminate the changing diffusion dynamics once we explicitly consider the network character of knowledge and compatibility-based learning. Building on this, Section 3.2 tackles the important topic of knowledge diversity within and between agents in an innovation network and the consequences for the performance of knowledge exploitation vs. exploration strategies. Thereafter, Sections 3.3 and 3.4 address the issue of measuring knowledge diffusion—while taking the three characteristics mentioned above seriously—both on an aggregated (Section 3.3) and an individual (Section 3.4) level. Finally, Section 3.5 contributes to discussions on the impact of an innovation network’s structural properties on the overall diffusion performance. In the last section of our article (Section 4), we draw our conclusion and suggest directions for future work.

## Theoretical background

### Relevant literature and research focus

Diffusion research can take various forms. It ranges from abstract and purely theoretical contributions to applied studies analyzing the effects of specific diffusion processes in detail. Any attempt to present a comprehensive overview would clearly go beyond the scope of this article.^2^

On a general note, we can, however, identify and differentiate four focus areas in the diffusion literature that address different questions, which are also important for this article’s focus on diffusion of knowledge:

- *what* diffuses?
- *how* does it diffuse?
- *where* does it diffuse?
- what are the *effects* (or performance) of the diffusion process and *how are they measured*?

To give just a brief summary, with regard to level ‘a) what diffuses’, methods and models range from capturing knowledge as simple information (e.g., numbers) or virus-like entities to more complex representations in terms of vectors, bit strings, or even graphs (see, e.g., Morone and Taylor 2010, for a review). The next important question is then, ‘how does it diffuse?’ (level b). This, of course, is to a certain extent also dependent on the representation of knowledge. Sometimes, diffusion exhibits features of *simple contagions* (such as infectious diseases), whereas on other occasions, one can observe *complex contagions*, which are affected by *homophily* and *social reinforcement* (Tur et al. 2014, 2018; Weng 2014; Weng et al. 2013; see also Lerman 2016). Moreover, this level concerns the way the diffusion process itself is designed. In some models, knowledge is exchanged via a barter trade mechanism, meaning that knowledge will only be exchanged if all partners involved can somehow benefit from the exchange (e.g., Cowan and Jonard 2004), whereas in others, knowledge may flow freely (see also Klarl 2014; Morone and Taylor 2009, on these different diffusion or transfer mechanisms). In addition, many of the models with an economic focus also incorporate the *absorptive capacity*^3^ of firms, which also influences the diffusion process (e.g., Cowan and Jonard 2004; Egbetokun and Savin 2014; Savin and Egbetokun 2016). For level ‘c) where does it diffuse?’, the modeling approaches to depict the underlying (social) structure range from diffusion or exchange on a grid or *von Neumann neighborhood* to complex network architectures (e.g., random, small-world, scale-free). Sometimes, models are also combined with real-world network data (e.g., Bogner et al. 2018) or with dynamic or (co-)evolving network structures (e.g., Luo et al. 2015; Tur and Azagra-Caro 2018). Alternatively, researchers have focused on the spatial dimension of knowledge diffusion (e.g., Canals et al. 2008). Finally, concerning the last level (d), the underlying question is how diffusion and its performance as well as effects are *measured*. This also differs between the various approaches, depending on the modelers’ focus. First, based on how knowledge is represented in level a, we could measure how much and how fast knowledge diffuses. This can be done either on an individual, i.e., *micro* level, or on a systemic *macro* level. Second, some models also shift the focus and measure “indirect” or economic effects of knowledge diffusion, e.g., in terms of increased returns, more product innovations, etc. (e.g., Cowan and Jonard 2007).

It is important to note, however, that these four levels can only to some extent be regarded in isolation. In fact, many models incorporate and analyze possible *feedback effects*. This means, for example, that—depending on how knowledge diffuses (level b) and on the effects of that process (level d)—other levels may be affected as well. In other words, the mechanisms and results of previous diffusion processes can also influence future performance either by changing an individual agent’s influence or importance (e.g., by becoming an *opinion leader* or a *gatekeeper*) or, at the systemic level, by dynamically changing the underlying social structure (level c). If the latter is represented as a network, diffusion impinges upon the network topology. This occurs, for instance, via link formation or deletion between the agents in the network, which in turn affects future diffusion (see, e.g., Canals 2005; Luo et al. 2015; Weng 2014; Tur and Azagra-Caro 2018).^4^

Nevertheless, at this point, our focus lies on the representation of knowledge (level a). As already mentioned above, previous approaches have modeled knowledge in different ways, all of which have particular merits and limits. Probably the simplest way to model knowledge is to assign a scalar number to each agent that represents the total amount of knowledge possessed by that agent. However, with such a simple approach it is impossible to differentiate qualitatively *what kind* of knowledge or information an agent holds. One way to tackle this problem is to model an agent’s knowledge as a vector, where each entry in the vector represents the “knowledge stock” of an agent in a particular knowledge *category*. Whenever an agent receives new knowledge related to a specific category, the respective number in the knowledge vector is increased (see, e.g., Bogner et al. 2018; Cowan and Jonard 2004; Luo et al. 2015; Mueller et al. 2017, for models using this knowledge representation). This approach already allows for a more qualitative view of knowledge while preserving its quantitative aspect, as the total knowledge is simply the sum of all entries in the vector. Nevertheless, it is not a priori clear if and how different knowledge categories are related to each other, and whether newly acquired knowledge can be relevant in several of such categories at the same time. Moreover, when knowledge is transferred to an agent, there is no way of determining whether the specific “kind” of received knowledge is already known to the agent or not. Consequently, there is no obvious way of representing how agents can learn *new* knowledge from each other, and thus, models incorporating such an approach usually assume that knowledge can only flow from those agents with a higher level of knowledge in a specific category to agents with a lower level in that category. Put differently, since numbers or vectors imply a rather easy quantifiability, they might obfuscate the complex nature of knowledge and learning, which often involves the creation of meaningful *connections* between ideas and concepts as well as their recombination (see also Arthur 2007; Markey-Towler 2016, 2017; Tywoniak 2007; Vermeulen and Pyka 2017, for related discussions). As Bernard Ancori and his coauthors explained almost two decades ago: “knowledge is not a mere stock resulting from the accumulation of an information flux” (Ancori et al. 2000, p. 259). In order to overcome this shortcoming, a representation of knowledge is required which ensures that what is learned is uniquely identifiable. One possibility to achieve this is a quantization of knowledge into distinct *units*, where each knowledge unit (KU) has a unique signature. The knowledge an agent possesses is then represented by a set of KUs.

Still, a simple set of KUs provides no notion of how these units may be related to each other. As Bart Nooteboom argues, one important implication of the approaches of *connectionism* and *parallel distributed processing* “is that knowledge is not stored in units, to be retrieved from there, but in patterns of activation *in connections between units*” (Nooteboom 2009, p. 51, emphasis added). We have, for this and other reasons explained below, decided to follow Paolo Saviotti (2009, 2011), who asserts that it is “possible to represent knowledge as a *network*” (Saviotti 2011, p. 151, italics in original). Therefore, in this work, we go one step further by connecting individual KUs based on a pairwise relation. Thus, the total knowledge an agent holds becomes a *network* of KUs.

### Motivation and foundations

Although we approach this complex endeavor by means of an abstract computational model, this article is motivated by—and rests upon—the theoretical background of *innovation systems* (see, e.g., Klein and Sauer 2016, for a review) and particularly *innovation networks* (e.g., Ahrweiler and Keane 2013; Buchmann and Pyka 2012; Koschatzky et al. 2001; Pyka and Küppers 2002). More precisely, we adopt the view of Tobias Buchmann and Andreas Pyka that the social structures where knowledge diffuses (our above level c) can be represented as innovation networks that “consist of actors and linkages among these actors. The idea of actors is conceived very broadly and also encompasses besides firms, individuals, research institutes and university laboratories, venture capital firms or even standardization agencies. Links among the actors are used as channels for knowledge and information flows ...” (Buchmann and Pyka 2012, p. 467).^5^

Since a novel way of representing knowledge (level a) also implies changes in how models capture its diffusion (level b), we require further theoretical foundations. Therefore, our approach also builds on the *memetics* literature, among others, especially when *memes* are understood as units of information transmitted primarily via social learning processes (e.g., Heylighen and Chielens 2009; von Bülow 2013, for an overview).^6^ In this sense, ideas or units of knowledge may also be conceived as memes “made” of (semantic) information (Dennett 1995, 2017) that can diffuse through or on social networks of agents (e.g., Gupta et al. 2016; Spitzberg 2014; Weng 2014).^7^ More importantly, the memetics literature also supports the idea of representing knowledge as a network based on the idea of *memeplexes* (e.g., Speel 1999), which may be conceived as complex systems or complex networks of memes that can replicate more successfully in an aggregated or connected form than the isolated memes on their own (Blackmore 1999; Heylighen and Chielens 2009; Schlaile 2018).

This element of interconnection and interdependence also relates to another important aspect of our approach, namely, that ideas, KUs, or memes need to have a certain *compatibility* with already existing ones: “An idea that is more compatible is less uncertain to the potential adopter and fits more closely with the individual’s situation. Such compatibility helps the individual give meaning to the new idea so that it is regarded as more familiar” (Rogers 2003, p. 240).^8^ In short: “knowledge requires knowledge to be assimilated” (Morone and Taylor 2010, p. 49, with reference to Ancori et al. 2000).

Note that the aspect of compatibility is also closely related to the concept of (optimal) *cognitive distance* (e.g., Boschma 2005; Nooteboom 1999, 2009; Nooteboom et al. 2007; Wuyts et al. 2005), because compatibility does not only mean similarity. The notion of cognitive distance implies that agents can only learn from each other and innovatively utilize the knowledge they exchange if their cognitions are neither too similar nor too different (see also Bogner et al. 2018). According to Nooteboom et al. (2007, p. 1017): “The challenge ... is to find partners at sufficient cognitive distance to tell something new, but not so distant as to preclude mutual understanding.” Indeed, this leads us to another aspect that distinguishes our approach from previous ones: Whereas cognitive distance can be conceived as a property at the agent level, compatibility focuses on the *knowledge level*. A network of compatible KUs or memes can thus be regarded as the shared mental representations necessary for constructing (interpretative) schemata, which in turn serve human agents as information filters (or: models *of* the world) as well as heuristics (or: models *for* the world) for discovering, creating, and exploiting opportunities (Schlaile and Ehrenberger 2016).

Another related strand of literature has argued that KUs or memes are faced with the “scarce resource” of *attention* (see also Weng et al. 2012).^9^ In line with Rogers (2003), Kate Distin explains: “Indeed, selection will often depend on a novelty’s *compatibility* with the rest of the meme pool. In their bid to gain and retain our *attention*, memes will succeed best if they fit in with facts and skills that we have already absorbed, being influenced particularly by those to which we are greatly attached” (Distin 2005, p. 205, emphasis added). As Herbert Simon famously argued, information consumes the attention of its recipients (Simon 1971), whereby it may be appropriate to speak of competition for attention among the nodes in the knowledge networks.^10^ Viewing attention as a scarce resource allows us to take up and integrate some approaches and notions from the *economics of attention* (e.g., Davenport and Beck 2001; Falkinger 2007, 2008): For the remainder of this article, we adopt the definition of attention proposed by Thomas Davenport and John Beck: “*Attention is focused mental engagement on a particular item of information*” (Davenport and Beck 2001, p. 20, italics in original). Note that in the case of organizational agents (as opposed to individuals), it may make sense to interpret the attention (i.e., ‘focused mental engagement’) of a firm or research institute more in terms of their *current focus*, e.g., in research and development (R&D). This aspect can be assumed to influence *how* knowledge diffuses (level b) and, therefore, also has to play an important role in our model below.

In summary, we propose a *network-of-networks* approach to capture knowledge diffusion and assimilation in innovation networks that also allows for the important issues of knowledge compatibility and scarcity of attention explained above.

### Model description

**Fig. 1:**
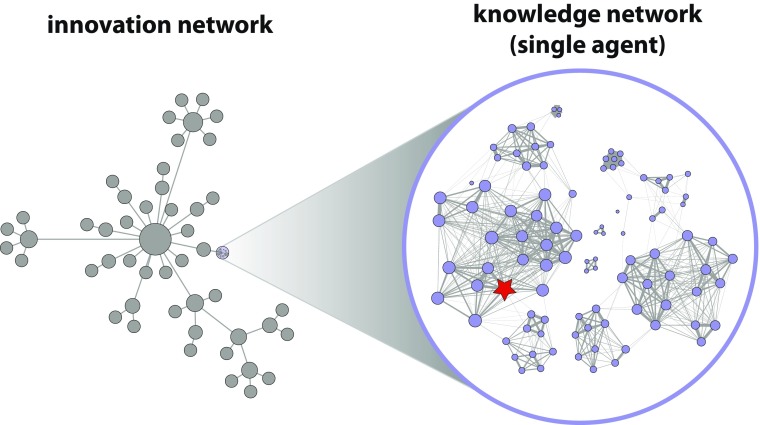
Illustration of the network-of-networks approach. The left side depicts an (arbitrary) innovation network *A* of agents {*a*_*i*_}, whereas the right side shows a magnification of a single agent’s knowledge network *B*_*i*_ with nodes {*b*_*ij*_} containing one KU *K*_*ij*_ per node. The currently focused KU is depicted as a red star

*Agent-based modeling* (ABM) has proven to be a useful method for simulating complex, dynamic phenomena (see, e.g., Gilbert 2008; Hamill and Gilbert 2016; Müller 2017; Schmid 2015; Wilensky and Rand 2015), including diffusion processes on networks (Garcia 2005; Kiesling et al. 2012; see also Barrat et al. 2008, or Namatame and Cheng 2016, for extensive reviews). As explained above, we employ a network-of-networks approach, meaning that each agent (e.g., firm or individual) $a_{i}$ in an innovation network $A = \left \{a_{i}|i\in \left [0,N_{A}\right )\right \}$ of size $N_{A}$ itself contains a network $B_{i} =\left \{b_{ij}|j\in \left [0,N_{B}\right )\right \}$ of size $N_{B}$ whose nodes $\{b_{ij}\}$ each contain a meme or knowledge unit $K_{ij}$ (see Fig. 1).^11^ Each $K_{ij}$ is in turn represented as a bit string of finite length $n_{K}$, where $n_{K}$ is fixed and identical for all $K_{ij}$ in the model.

Incorporating the aspects of knowledge compatibility and scarcity of attention in our model also affects the way knowledge is exchanged and thereby diffuses through the network of agents. Instead of passively exchanging KUs that just “improve” the knowledge stock of its recipients, new KUs now have to be assimilated and integrated into the existing knowledge network, taking into account that the construction and diffusion of knowledge is a path-dependent process (e.g., Baddeley 2010; Rizzello 2004, with reference to Hayek 1952). As explained in the previous section, successful assimilation of new KUs into the existing knowledge network will be highly dependent on *compatibility* with prior / existing knowledge of the recipient.^12^ This aspect represents another important novelty of our model: In several previous models, the absorptive capacity of firms is an exogenous model parameter (e.g., Cowan and Jonard 2004; Mueller et al. 2017), whereas in our model, a firm’s ability to recognize the value of new, external knowledge and assimilate it is endogenous, emerging from the compatibility of the received KUs with the agent’s existing knowledge network.^13^ Our model captures this central aspect by introducing a measure of compatibility *c* between KUs based on their normalized *Hamming distance* *d* (Hamming 1950). It is defined as

1 $$ c_{jk}\equiv c(K_{ij}, K_{ik}) = 1 - d(K_{ij}, K_{ik})\,\in[0,1]\,, $$

where the KUs $K_{ij}$ and $K_{ik}$ are bit strings of fixed length $n_{K}$. The normalized Hamming distance *d* can then be expressed as

2 $$ d_{jk}\equiv d(K_{ij}, K_{ik}) = \frac{1}{n_{K}}\sum\limits_{m = 0}^{n_{K}-1}\left[K_{ij}\oplus K_{ik}\right]_{m}\,, $$

where $\oplus $ denotes the logical bitwise XOR (“exclusive or”) operator, while the operator $[\cdot ]_{m}$ returns the *m*-th bit of its operand.

The model is set up as follows: First, the agent network *A* is filled with $N_{A}= 100$ agents $a_{i}$, and $M_{A}= 200$ undirected edges are established between pairs of agents according to a user-selected type of network, or loaded from an existing structure. Each of the agents’ knowledge networks $B_{i}$ is then filled with $N_{B}= 100$ nodes $b_{ij}$ (unless stated otherwise), and each $b_{ij}$ receives a unique KU $K_{ij}$ of length $n_{K}= 32$ bits. All pairs of nodes $\left (b_{ij}, b_{ik}\right ),\,j\neq k$ within an agent $a_{i}$’s knowledge network $B_{i}$ are then connected by an undirected edge $e_{i(j,k)}$ if the condition $P_{jk}= 1$ is fulfilled, where $P_{jk}$ is a function of the respective KUs’ compatibility:

3 $$ P_{jk}\equiv P(c_{jk}) = \left\{\begin{array}{ll} 1 & \text{if}\ \gamma < c_{jk} < 1\\ 0 & \text{otherwise} \end{array}\right. $$

Each of the edges $\left \{e_{i(j,k)}\right \}$ created in this manner is given an edge weight $w_{i(j,k)}=c_{jk}$. The rectangular “compatibility window” described by Eq. 3 is chosen solely due to its simplicity, and could in principle be of any other suitable shape. Nevertheless, the threshold $\gamma $ has a distinct meaning: We interpret a compatibility of $c_{jk}= 0.5$ as a point of indifference, dividing the compatibility range qualitatively into the lower half with $c_{jk}<0.5$, where the respective KUs $K_{ij}$ and $K_{ik}$ are believed to be rather incompatible, and the upper half with $0.5<c_{jk}$, where they are considered to be rather compatible. According to this interpretation, $\gamma $ should be greater than 0.5. However, the compatibility measure is based on the normalized Hamming distance, which in turn is also a measure of *similarity*. Thus, a compatibility of $c_{jk}= 1$ implies $K_{ij}\equiv K_{ik}$. The condition $P_{jk}$ will also be used to determine the exchange of KUs between agents, and since firms or individuals are usually not interested in learning what they already know, $c_{jk}$ must be strictly smaller than one to fulfill $P_{jk}= 1$. Note that, as already mentioned above, the idea of compatibility is related to the notion of (optimal) cognitive distance. By introducing the condition $P_{jk}$, we are in line with Nooteboom and others (for an overview, see, e.g., Nooteboom 2009) who argue that learning and innovative performance depend on the agents being able to learn something new while at the same time making sense of the knowledge they exchange. In other words, agents’ cognitions should not be too distant from their partners’. By means of our compatibility window, we apply these ideas to the level of the KUs.

Now that the initial setup of the model is complete, we proceed by defining the rules governing its temporal progress. As already explained in the previous section, several scholars have argued that knowledge and information consume the attention of their recipients, resulting in some kind of competition among the KUs for the “scarce resource” of attention (e.g., Simon 1971; Weng 2014; Weng et al. 2012). Moreover, in the context of firms or research institutes, we have proposed that this “attention” could be interpreted as their current *R&D focus*. Our model incorporates shifts of this focus as a weighted random walk from node to node along the existing edges of the agent’s knowledge network, starting at a randomly chosen node.

**Fig. 2:**
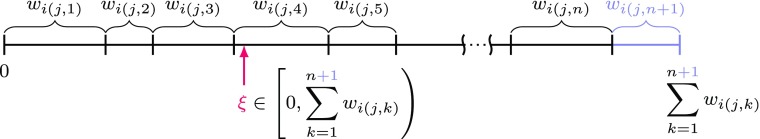
Illustration of the selection mechanism used in the random walk along the edges in a knowledge network *B*_*i*_. Let *b*_*ij*_ be the currently focused node with degree *n* in the knowledge network *B*_*i*_. The cumulative sums of the weights of edges connecting *b*_*ij*_ with its *n* neighbors are stored. Additionally, a virtual edge to a randomly chosen node from *B*_*i*_ is considered and its corresponding edge weight is added to the cumulative sums in order to allow random jumps to arbitrary nodes in the network. Then, a random number *ξ* is uniformly chosen on the interval $\left [0,{\sum }_{k = 1}^{n + 1}w_{i(j,k)}\right )$. The index *k* of the interval to which *ξ* corresponds is determined, and the random walk is advanced to the thereby chosen *k*-th neighbor node of *b*_*ij*_

Thus, at every time step *t*, in ABM often referred to as a *tick*, the focus of each agent $a_{i}$ changes its position in the agent’s knowledge network $B_{i}$ by advancing from the currently focused node $b_{ij}$ to an adjacent node $b_{ik}$. Since a node $b_{ij}$ usually has more than one adjacent neighbor node, the probability of advancing to any particular adjacent node $b_{ik}$ is weighted by the connecting edge’s weight $w_{i(j, k)}$. The procedure of selecting the next node in the random walk is depicted in Fig. 2.

As the weighted random walk depicts the agents’ shifting focus in their respective knowledge networks, in the case of organizational agents (e.g., firms), this weighted random walk can also be interpreted as a measure for the agent’s strategy of knowledge *exploitation* vs. *exploration* (see also the discussion in March 1991; Schmid 2015).^14^ For our model, this means that in the latter case, the focused KU would be picked randomly with uniform probability, i.e., without taking the structure of the knowledge network into account. To distinguish the two cases, we will refer to them as (weighted) random walk and (unweighted) random jump, respectively.

Diffusion of knowledge in the innovation network *A* is realized by communication between pairs of agents according to a simple *knowledge transfer protocol*. For technical reasons and for the sake of a meaningful analysis by focusing on our knowledge representation as a network (level a), at this stage, we chose not to impose any restrictions on how knowledge diffuses (level b) aside from compatibility.^15^ Therefore, the trade protocol chosen at this stage is a *knowledge pull* mechanism with high fidelity (i.e., there are no errors during replication): At every tick (after each step of the random walk), each agent randomly selects *one* of its neighboring agents and retrieves this agent’s currently focused KU. The compatibility of this KU with the receiving agent’s currently focused one is then evaluated, and, if these two KUs satisfy the condition given in Eq. 3, the received KU is integrated into the receiving agent’s knowledge network. The integration of such a newly received KU follows the very same procedure as applied during the construction of the initial knowledge network, i.e., edges are established to all previously existing KUs if their compatibility *c* with the new unit satisfies the condition *P*(*c*) = 1 according to Eq. 3. By choosing this “instantaneous” way of integrating a new KU into a knowledge network, we implicitly make the assumption that the internal communication within each agent is much faster than the external communication between different agents. Further details on the choice of parameters and the technical framework are given in Appendices A and B.

## Results

### Baseline analysis

In order to investigate the effects of compatibility on the dynamics of knowledge diffusion in our model, we first take a step back and eliminate the influence of compatibility completely. This is accomplished by setting the compatibility threshold $\gamma $ to zero, so that agents can take up any kind of new KUs. Furthermore, we switch off the weighted random walk along the edges in the agents’ knowledge networks, which means that an agent’s focus can jump to any of its KUs with equal probability. Effectively, this implies that, for a brief moment, we go back to a model where an agent’s knowledge is not represented by a network but by a set of unconnected KUs.

If we went back even further and did not consider the *uniqueness* of KUs, i.e., agents could take up KUs they already have, the resulting dynamics would be trivial: Since, then, every agent is allowed to retrieve one KU from a neighboring agent per time step, the number of KUs per agent would simply increase linearly with time. This also means that it would be impossible in this case that the topology of the innovation network *A* could have any effect on knowledge exchange. In that respect, we want to stress that this simple thought experiment justifies our choice of knowledge trade mechanism, since a simple knowledge pull ensures the elimination of topological effects that are not based on knowledge compatibility.

Starting from this point, we will now continue by successively incorporating more and more features of our model and study their effects.

First, we only consider the uniqueness of KUs and leave the other features of our model turned off. If agents only take up KUs that are new to them, the maximum number of KUs an agent can assimilate is limited to the total number of unique KUs existing in the simulated system, and growth is bounded. Initially, the probability of retrieving a new KU is generally high, and thus, growth is fast. However, the more KUs an agent has, the less likely it is for that agent to retrieve new KUs. Therefore, the growth rate decreases over time.

**Fig. 3:**
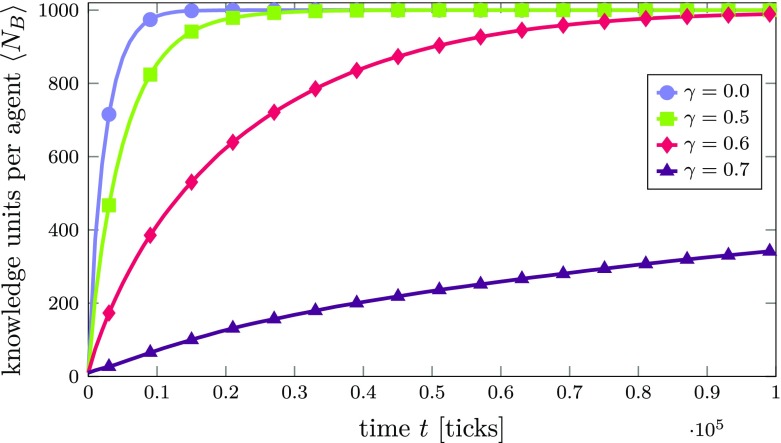
Average number of KUs $\left <N_{B}\right >$ per agent over time in an innovation network *A* with an Erdős-Rényi (random network) topology for different compatibility thresholds *γ*. Lines are averages over 100 agents. The standard error of the mean is smaller than the line width and therefore not shown

If we now successively increase the compatibility threshold $\gamma $ while still leaving the random walk switched off (i.e., all agents can be imagined to follow a “knowledge exploration” strategy, where focused attention on any of the agent’s KUs is equally likely), the probability to retrieve new knowledge that is *compatible* to an agent’s currently focused KU decreases with increasing $\gamma $. Figure 3 shows the average number $\left <N_{B}\right >$ of KUs per agent over time for different values of $\gamma $.^16^

The observed effect of an increasing compatibility threshold $\gamma $ is that the time scale of the simulation is simply stretched, since the growth rate of an agent’s knowledge is decreased with increasing $\gamma $.^17^ When relating this result to a real-world learning process, it trivially means that if it is harder for an agent to find compatible knowledge, learning will take more time.

As a next step, we will establish the actual representation of an agent’s knowledge as a network $B_{i}$ of KUs $\{K_{ij}\}$, which are connected by edges $\{e_{i{(j,k)}}\}$ based on their pairwise compatibility $c_{jk}$ (1) under the $\gamma $-dependent condition $P_{jk}$ (3). This allows us to switch on the weighted random walks of the agents’ shifting attention or focus along these edges, with the consequence that KUs that are strongly connected to other related KUs have a higher probability to attract attention, and, therefore, also a higher probability of being transmitted.

**Fig. 4:**
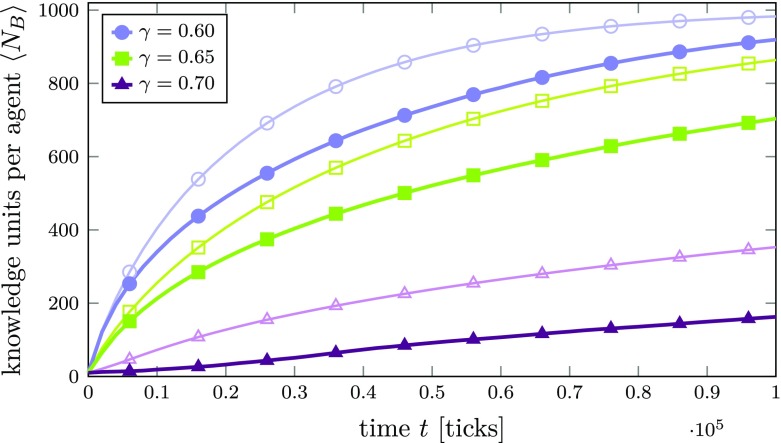
Average number of KUs $\left <N_{B}\right >$ per agent over time in an innovation network *A* with an Erdős-Rényi (random network) topology for different compatibility thresholds *γ*. Thick lines with filled symbols are obtained from simulations with the random walk within the agent’s knowledge networks switched on, while thin lines with corresponding empty symbols and colors are taken from simulations with unweighted random jumps. Lines are averages over 100 agents. The standard error of the mean is smaller than the line width and therefore not shown

As above, Fig. 4 shows the average number $\left <N_{B}\right >$ of KUs per agent over time for different values of $\gamma $. Curves with thick lines and filled symbols are obtained from simulations with the weighted random walk within the agent’s knowledge networks switched on (knowledge exploitation strategy), while thin lines with corresponding empty symbols and colors are taken from simulations with unweighted random jumps (knowledge exploration strategy).^18^

The first and obvious observation clearly is that the curves from simulations with knowledge exploitation (i.e., random walk switched on; thick lines with filled symbols) generally lie below the corresponding curves obtained from simulations where all agents follow the exploration strategy (i.e., random jumps; thin lines, empty symbols, same color code). This indicates that the exploitation strategy of agents focusing on well-connected KUs (i.e., those exhibiting strong compatibility relations with others within the agents’ knowledge networks) causes a slow-down in the dynamics of knowledge diffusion between different agents. More precisely, this will cause neighboring agents to exchange knowledge only in a limited field, thereby successively decreasing their chance that they can learn something new, causing their attention to be “paradigmatically locked in” in a truly Kuhnian sense.^19^

Moreover, the average growth rates now exhibit a slightly different time dependence compared to the case with random jumps (exploration).^20^ While at the very beginning of the simulation the curves for $\gamma = 0.60$ and $\gamma = 0.65$ (blue lines with squares and green lines with circles, respectively) almost coincide with the corresponding curves from simulations with random jumps, they quickly start to deviate. In contrast to those two cases, for $\gamma = 0.7$, the beginning of the curve with the random walk switched on (thick purple line, filled triangles) is convex. As we will see at a later point in this work, this specific behavior originates from the fact that some agents in the simulation may be unable to find any compatible knowledge among their neighbors in the early stages of the simulation if the compatibility threshold is rather high.

### Effects of knowledge diversity

It has been observed that innovation networks, sectors, regions, or industries often exhibit different and uneven developments also in terms of knowledge, whereby it becomes increasingly important to better understand the factors contributing to these diverging knowledge trajectories (e.g., Feldman and Audretsch 1999; Foray 2004 2014; Frenken et al. 2007; Smith 2000; Vermeulen and Pyka 2017). Therefore, in this section, we extend our baseline model to analyze if and how the (initial) diversity of knowledge bases in an innovation network can influence the knowledge diffusion process.

So far, we have only considered systems where KUs originate from the same uniform distribution for all agents in the innovation network. Even though, for this setup, the dependence of knowledge exchange on compatibility as well as the introduction of the random walk results in a statistically significant quantitative change of knowledge diffusion dynamics, the qualitative behavior is still very similar to the case where compatibility has no influence on knowledge exchange at all. As we will see in the following subsections, this will change drastically if we consider different levels of knowledge homo- and heterogeneity within and among agents.

In the following two paragraphs, we construct two different scenarios of knowledge diversity. In the first scenario, we imagine an example where all agents essentially share the same knowledge background, for example, because they are rooted within the same technological field. In other words, in this first scenario, all agents’ knowledge networks share the same “ancestral” knowledge unit (AKU). In the second scenario, we consider a situation where agents do not share a common AKU, for example, because the innovation network consists of agents from different technological fields.

#### Common knowledge background

**Fig. 5:**
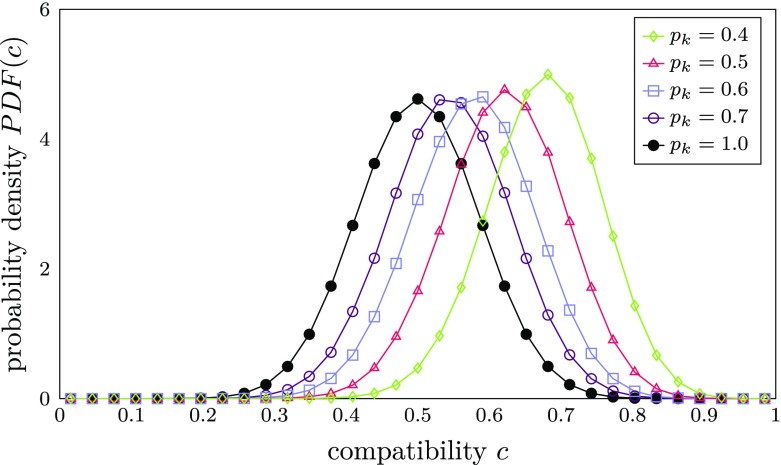
Distribution of compatibilities between pairs of KUs with *n*_*K*_ = 32 bits generated from an AKU with different bit reassignment probabilities *p*_*k*_. The area under the curves is normalized to 1 so that the curves can be interpreted as probability density functions (PDFs). The case *p*_*k*_ = 1 (black line with filled circles) corresponds to the case where each knowledge unit is completely random and independent of the AKU

The representation of KUs as bit strings offers the possibility to control their average pairwise compatibility by employing different methods of bit string generation. If all KUs are generated randomly from a uniform distribution, the distribution of compatibilities between all pairs of such KUs has the shape of a Gaussian centered at $c = 0.5$. If the integral of the compatibility distribution is normalized to 1, the distribution can be interpreted as a probability density function *PDF*(*c*) (see black line with filled circles in Fig. 5 below). Obviously, this distribution remains intact during the entire course of the simulation, since each newly acquired KU originates from the same initial uniform distribution.

In order to implement the scenario where agents have a common knowledge background, the average pairwise compatibility $c_{mn}$ between pairs of KUs $K_{im}$, $K_{jn}$ belonging to any two different agents $a_{i}$, $a_{j}$ has to be shifted closer to one. This can be accomplished by initially supplying all agents in the system with the same AKU (arbitrarily chosen for each simulation run during initialization). Each KU of an agent is then derived from a copy of the AKU by reassigning a random value to each bit of the copied AKU with a reassignment probability $p_{k}$. Accordingly, for $p_{k}= 0$, no bits would be changed and, thus, all KUs would equal the AKU. In contrast, for $p_{k}= 1$, each KU would be drawn from a uniform random distribution again, which would lead to the same overall compatibility distribution as before. To illustrate this behavior, Fig. 5 depicts compatibility distributions resulting from different values of *p*_*k*_.

Since the AKU is the same for each agent in *A*, these compatibility distributions represent compatibilities between all pairs of KUs within the whole simulated system, i.e., between and within agents. Consequently, a lower value of $p_{k}$ implies a higher knowledge homogeneity in the system.

**Fig. 6:**
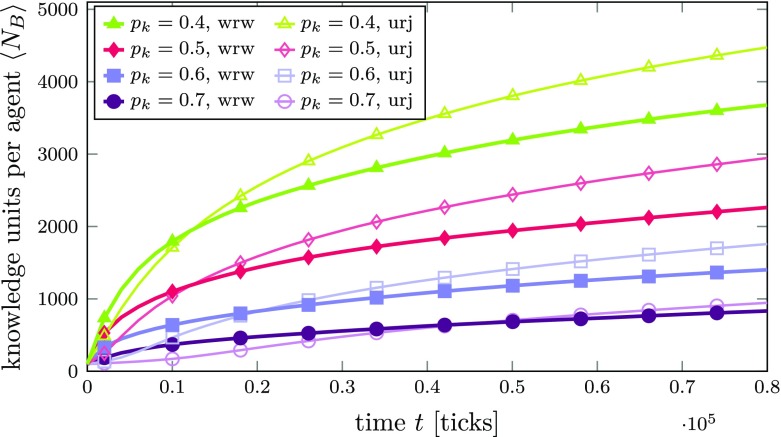
Average number of KUs $\left <N_{B}\right >$ per agent over time in an innovation network *A* with an Erdős-Rényi (random network) topology for different KU bit reassignment probabilities *p*_*k*_. The compatibility threshold was set to *γ* = 0.75 in all simulations. Thick lines with filled symbols are obtained from simulations with the random walk within the agent’s knowledge networks switched on (wrw), while thin lines with corresponding empty symbols and colors are taken from simulations with unweighted random jumps (urj). Lines are averages over 100 agents. The standard error of the mean is smaller than the line width and therefore not shown

Figure 6 shows the diffusion performance, again measured by the average number of KUs per agent over time. As before, we compare the results for exploitation and exploration strategies (i.e., with and without our weighted random walk). The first observation is that, for higher knowledge homogeneity (systems with lower $p_{k}$), the learning process is much faster since it is easier for the agents to find compatible knowledge. The second observation is that, in contrast to the previous case (Fig. 4), for all depicted levels of knowledge heterogeneity, initially a knowledge exploitation strategy (weighted random walk on) means that learning is faster but, after a certain point in time (earlier for lower $p_{k}$), knowledge exploration becomes more efficient. If an agent focuses on a highly compatible subset of its knowledge, the average compatibility with such a subset focused by another agent is comparatively high, since agents have a common knowledge background. KUs belonging to these subsets can therefore be transferred very rapidly at the beginning. Consequently, we see that the “paradigmatic lock-in” described above may actually be beneficial at the beginning of the diffusion process if agents share a common knowledge background. However, we can still observe that this lock-in caused by exploitation leads to a situation where knowledge diffusion levels off and, thus, in the long run, exploration will be more conducive to knowledge diffusion.^21^

#### Different knowledge background

In contrast to the previous case, we now consider a scenario where we no longer assume a common knowledge background for all agents in the system, for example, because actors in the innovation network come from different technological fields. In the model, instead of deriving each knowledge network from *the same* AKU, agents derive their knowledge networks from *diverse* AKUs now.

We achieve this diversity in knowledge background by reassigning a random value to each bit of an agent’s AKU with an AKU bit reassignment probability of $p_{a}$ while keeping the KU bit reassignment probability constant at $p_{k}= 0.6$. This means that for $p_{a}= 0$, we would have the same scenario as before, and each agent would have the same AKU, whereas for $p_{a}= 1$, each agent has a random AKU. More precisely, in our example, $p_{k}$ can be interpreted as a measure for the heterogeneity of knowledge within a particular technological field, whereas $p_{a}$ determines the heterogeneity across technological fields within an innovation network. Since $p_{k}$ is fixed to 0.6 in this scenario, increasing $p_{a}$ essentially means increasing the average cognitive distance between agents.

**Fig. 7:**
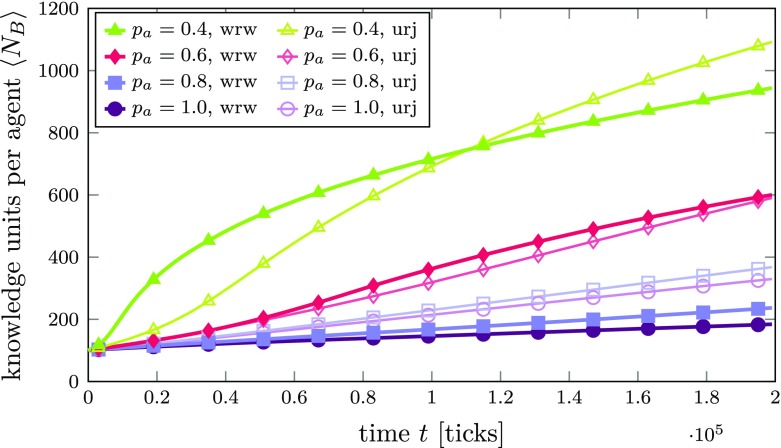
Average number of KUs $\left <N_{B}\right >$ per agent over time in an innovation network *A* with an Erdős-Rényi (random network) topology for different AKU bit reassignment probabilities *p*_*a*_. The KU bit reassignment probability was set to *p*_*k*_ = 0.6 and the compatibility threshold to *γ* = 0.75 in all simulations. Thick lines with filled symbols are obtained from simulations with the random walk within the agent’s knowledge networks switched on (wrw), while thin lines in corresponding colors with empty symbols are taken from simulations with unweighted random jumps (urj). Lines are averages over 1,000 agents. The standard error of the mean is smaller than the line width and therefore not shown

In Fig. 7, we show the results for different degrees of average cognitive distance, ranging from $p_{a}= 0.4$ to $p_{a}= 1.0$. As above, the curves depict the (average) growth of agents’ knowledge networks over time. The first and probably most obvious thing to observe is that learning is much slower and the agents’ average number of KUs considerably lower than in the previous scenario with the common knowledge background.^22^ Yet, the most striking result compared to the previous case is that for higher $p_{a}$, i.e., for situations where the AKUs of agents differ considerably (*p*_*a*_ = 0.8 and $p_{a}= 1.0$), knowledge exploration is always “better” in terms of knowledge network growth than knowledge exploitation. However, for moderate levels (*p*_*a*_ = 0.4 and $p_{a}= 0.6$), we see that exploitation leads to a faster learning process in the short and medium term. In other words, introducing different technological fields within a network—and hereby increasing the initial diversity of knowledge between agents—does not lead to a situation where an exploration strategy is always advantageous. Instead, we see that exploitation is more beneficial (at least in the short and medium term) in networks with relatively compatible but still different technological fields.

### Extended analysis: approaching the qualitative dimension of knowledge

Up to this point, we have restricted ourselves to assessing overall quantitative changes in terms of the average number of KUs per agent, which gives us a measure for successful knowledge diffusion and, especially, assimilation processes. However, by changing the way knowledge representation is modeled from numbers and vectors to networks, we have arrived at a point where we can also re-conceptualize how the *effects* of knowledge diffusion are analyzed. As our model allows us to not just deal with agents’ higher or lower knowledge “stocks” or knowledge “levels”, we can shift the focus from cardinal measures referring to “more” knowledge, to an extended, qualitative, i.e., structural, analysis at the knowledge network level (*B*_*i*_). A large number of different approaches and indices exist to describe a network’s structural properties (e.g., Barabási 2016; Newman 2010; Wasserman and Faust 1994). However, although well-established for the description of social and other complex networks, an application to knowledge networks is not straightforward, particularly because the knowledge networks in our simulation are dynamic and may contain disconnected components, whereas many measures can only be meaningfully applied to static networks and connected graphs. Moreover, some measures such as the average path length are size-dependent and rather meaningless for growing knowledge networks with disconnected components, whereas other measures such as clustering coefficients are hard to interpret.^23^ Nevertheless, there exist some network characteristics that can be used to illustrate the merits of our model.

In the following analysis of the structure of knowledge networks, we focus on the number of KUs, average degree, (weighted) density, and modularity of the knowledge networks. As before, the number of KUs is an important measure for knowledge diffusion performance. The average degree of KUs is, of course, also size-dependent and indicates the number of KUs in that network with which an average KU is compatible. The weighted density of a knowledge network indicates how well an agent can, on average, put its KUs into context. Modularity is a measure used for community detection in networks (e.g., Francisco and Oliveira 2011; Newman 2004a, 2004b; Newman and Girvan 2004; Sobolevsky et al. 2014). In the case of our model’s knowledge networks, modularity may be a reasonable way to capture an agent’s relative degree of knowledge *diversification* (high modularity) or *specialization* (low modularity in conjunction with high weighted density).^24^

**Fig. 8:**
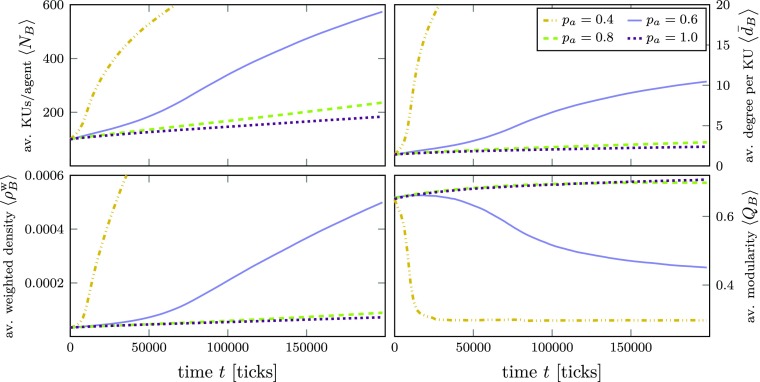
Change of knowledge network properties over time in an innovation network *A* with an Erdős-Rényi (random network) topology for different AKU bit reassignment probabilities *p*_*a*_. The lines are averages over 1000 agents. The KU bit reassignment probability was set to *p*_*k*_ = 0.6 and the compatibility threshold to *γ* = 0.75 in all simulations. Knowledge exploitation by all agents (wrw). Left: Average number $\left <N_{B}\right >$ of KUs in {*B*_*i*_} and average weighted network density $\left <\rho ^{\mathrm {w}}_{B}\right >$ of {*B*_*i*_}. Right: Average degree $\left <\bar {d}_{B}\right >$ of KUs in {*B*_*i*_} and average modularity $\left <Q_{B}\right >$. The standard error of the mean is ± 1.5 ⋅ 10^− 5^ for $\left <\rho ^{\mathrm {w}}_{B}\right >$ and in the order of the line width for the other three properties

Figure 8 shows the results for these indicators as an average over 10 simulation runs (i.e., 1,000 agents) for different degrees of initial cognitive distance (varying $p_{a}$). In this scenario, all agents pursue knowledge exploitation (weighted random walk on). As we have already seen above (in Fig. 7), learning is slower for higher $p_{a}$. Consequently, the average degree and the weighted density also increase much faster in the case of lower average cognitive distance, as it is harder for more diverse agents to “make sense” of the knowledge they receive from agents with a different knowledge background. Although all agents engage in knowledge exploitation (weighted random walk), by looking at the modularity we can see that in cases where average cognitive distance is lower (smaller $p_{a}$), modularity decreases, pointing to a tendency to specialize around a particular field, whereas for higher average cognitive distances (higher $p_{a}$), modularity increases and points to a more “clustered” or diversified knowledge base.^25^

### The importance of being in the right place at the right time

**Fig. 9:**
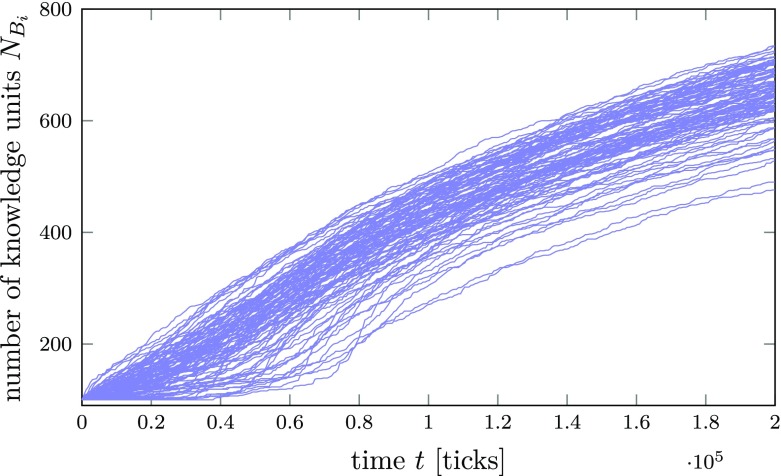
Change of individual knowledge network sizes $N_{B_{i}}$ over time in an innovation network *A* with an Erdős-Rényi (random network) topology for a moderate level of cognitive distance (*p*_*a*_ = 0.6 and *p*_*k*_ = 0.6), knowledge exploitation by all agents and *γ* = 0.75

Although researchers and policy-makers may often be interested in the overall “performance” of a particular region or system (see, e.g., Foray 2014; Vermeulen and Pyka 2017), it should be kept in mind that the simulation results shown above (e.g., Fig. 8) are averaged over 10 simulation runs (i.e., 1000 agents). However, by averaging time series of dynamic quantities, many details of the underlying dynamics might be obscured. In order to illuminate more details of individual knowledge network dynamics, we therefore show the individual time series for the number of KUs of each agent in one simulation in Fig. 9.

As we can see, agents’ learning trajectories differ. In fact, at the beginning of the simulation, there are agents who are unable to find any compatible knowledge. This results in the convex shape of some of the averaged curves in Figs. 6–8. To investigate the origins of these individual learning differences, we looked at the dependence of the individual agents’ knowledge networks on their local degree, clustering coefficient, betweenness centrality, and harmonic closeness centrality. We found that a higher degree in *A* has a positive impact but with decreasing returns to scale. Harmonic closeness centrality was found to have the strongest impact, followed by degree and betweenness centrality. In contrast, an agent’s local clustering coefficient was found to have only a small but negative impact on an agent’s learning performance.^26^

**Fig. 10:**
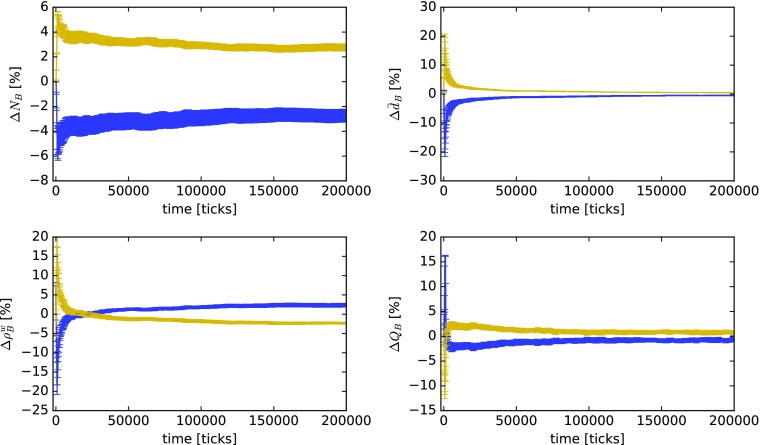
Time series of relative deviations from the mean of different knowledge network properties depending on agents’ harmonic closeness centrality *H* in the innovation network *A* for the case of a common knowledge background (*p*_*a*_ = 0.0) and a moderate level of knowledge heterogeneity (*p*_*k*_ = 0.6). Investigated properties of the agents’ knowledge networks are size *N*_*B*_ (top left), average degree $\bar {d}_{B}$ per KU (top right), weighted density $\rho ^{\mathrm {w}}_{B}$ (bottom left), and modularity *Q*_*B*_ (bottom right). Yellow lines show averages for agents belonging to the group with *H* above the median and blue lines for agents belonging to the group with a value of *H* lying below the median. The data were computed from a system of 100 agents. Knowledge exploitation by all agents and *γ* = 0.75. Error bars represent the standard error of the mean of each group

As an example, Fig. 10 shows the time series of relative deviations from the mean of different properties in $B_{i}$ depending on agents’ harmonic closeness centrality *H* in *A* in a scenario where all agents have a common knowledge background (*p*_*a*_ = 0) and engage in knowledge exploitation (weighted random walk). For the analysis, we first categorize agents into two groups, one containing agents with *H* above the median and the other containing agents with *H* below the median. Second, we compute the average relative deviations for each group from the mean of the entire population for the different properties of $B_{i}$ mentioned before (*N*_*B*_, $\bar {d}_{B}$, $\rho ^{\mathrm {w}}_{B}$, and $Q_{B}$) according to

4 $$ {\Delta} X = \frac{1}{g}\sum\limits_{i = 1}^{g}\frac{X_{i}-\left<X\right>}{\left<X\right>}\,, $$

where $X\in \{N_{B},\,\bar {d}_{B},\,\rho ^{\mathrm {w}}_{B},\,Q_{B}\}$, *g* is the number of agents belonging to one of the groups (below or above median *H*), and the operator $\left <\cdot \right >$ denotes the average over all agents in the simulation, regardless of their group membership. Note that, according to Eq. 4, the different ${\Delta } X$ reported in the subgraphs of Fig. 10 are *group averages* of the relative deviation from the mean of the entire population.

In Fig. 10, the large positive and negative peaks of ${\Delta } \bar {d}_{B},\,{\Delta } \rho ^{\mathrm {w}}_{B}, $ and ${\Delta } Q_{B}$ at the beginning of the simulation are due to a combination of two things: First, agents with a high value of *H* are, on average, more likely to also have a higher degree. This means that they have a higher probability of finding compatible knowledge among their neighbors and can learn more rapidly. Second, knowledge networks are initially very small (100 KUs), with the effect that an additional KU can significantly reduce the modularity of the knowledge network. Consequently, since we are dealing with *relative* deviations from the mean, the curves of the other group (with low *H*) show the opposite behavior. In summary, the data presented in Fig. 10 imply that if the agents in an innovation network with moderate levels of knowledge heterogeneity (*p*_*k*_ = 0.6) have a common knowledge background (*p*_*a*_ = 0), in the long run, short communication paths to the other agents in the network (higher *H*) result in a small but almost constant advantage in learning with slightly higher knowledge diversification.

**Fig. 11:**
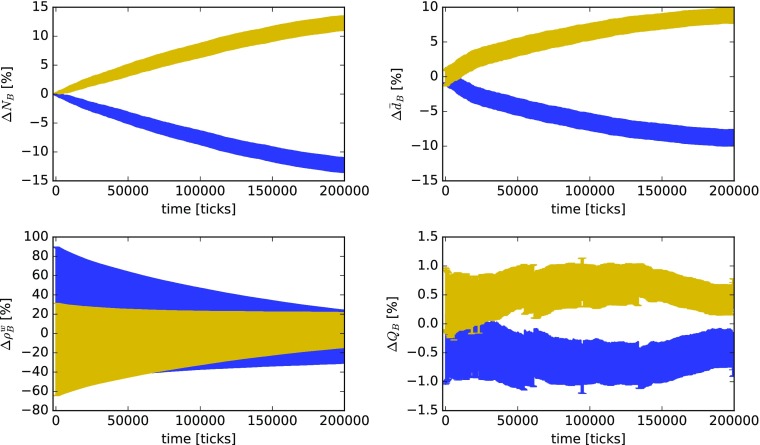
Time series of relative deviations from the mean of different knowledge network properties depending on agents’ harmonic closeness centrality *H* in the innovation network *A* for the case of highly diverse knowledge backgrounds (*p*_*a*_ = 0.8) and a moderate level of knowledge heterogeneity (*p*_*k*_ = 0.6). Investigated properties of the agents’ knowledge network are size *N*_*B*_ (top left), average degree $\bar {d}_{B}$ per KU (top right), weighted density $\rho ^{\mathrm {w}}_{B}$ (bottom left), and modularity *Q*_*B*_ (bottom right). Yellow lines show averages for agents belonging to the group with *H* above the median and blue lines for agents belonging to the group with a value of *H* lying below the median. The data were computed from a system of 100 agents. Knowledge exploitation by all agents and *γ* = 0.75. Error bars represent the standard error of the mean of each group

Figure 11 also shows the time series of average relative deviations from the mean of different properties in $B_{i}$ depending on *H* in *A*; however, this time for a scenario with highly diverse knowledge backgrounds (*p*_*a*_ = 0.8; all other parameters are equal to the previous case presented in Fig. 10). As we can see, in the long run, in innovation networks with agents from initially highly different knowledge backgrounds, “closer” agents (with *H* above the median) have, on average, a significant advantage in the number of KUs and their KUs are also more compatible to others in their knowledge network (higher $\bar {d}_{B}$). In other words, in innovation networks with actors from diverse knowledge backgrounds, agents with fewer and longer communication paths have a higher chance that incompatible (i.e., cognitively distant) agents effectively block their transmission of compatible knowledge. In contrast, learning processes of rather well-connected agents with high *H* are less likely to be “stalled” by other agents. Finally, in Fig. 11, modularity does not show significant differences, with the implication that—although advantageous for learning—shorter communication paths do not seem to influence an agent’s propensity for knowledge diversification or specialization.

### Results for different innovation network properties

As we have found that the agents’ local properties or positions in the innovation network *A* can influence their learning processes, we also expect the global properties of *A* to have an impact on overall diffusion performance. This is in line with many models of knowledge diffusion on networks that have, for various reasons, focused on analyzing the speed and extent of the diffusion process depending on different topologies of the agents’ social network (e.g., Bogner et al. 2018; Buchmann and Pyka 2012; Cowan and Jonard 2004; Morone et al. 2007; Mueller et al. 2014, 2017; among others). In this section, we therefore analyze different (static) structures of *A* and compare the dynamics at the knowledge network level (*B*_*i*_) depending on different types or properties of *A*.^27^

Up to this point, innovation networks in our analyses were assumed to be an Erdős-Rényi (ER) random graph (Erdős and Rényi 1959, 1960). Here, we additionally use the other two most widely-used network types of Barabási-Albert (BA) (Barabási and Albert 1999, 2002) and Watts-Strogatz (WS) (Watts and Strogatz 1998) to compare the resulting dynamics in $B_{i}$ for different structures of *A*.

As already mentioned above, for the sake of comparability, we fix the number of agents and the number of links between them for all network types. We additionally require the networks to not have any disconnected components.^28^ Table 1 shows how the networks differ in their properties. Judging from our previous results, we expect networks with a high average harmonic closeness centrality *H* and a low clustering coefficient to perform best in terms of knowledge network growth. Additionally, since an agent’s local degree showed “decreasing returns to scale”, networks with a high median degree should be advantageous for knowledge diffusion. If we compare the properties of the different types of *A* listed in Table 1, we expect the following: Both BA and ER have a relatively high *H* and relatively low clustering coefficients as well as comparatively small network diameters and should, therefore, facilitate knowledge diffusion more efficiently than the WS network, so that the expected “ranking” is ER $\approx $ BA $>$ WS. However, due to our focus on compatibility-based learning and the simple knowledge trade protocol we employ, we do not expect to see fundamental qualitative differences in the average diffusion performance. Nevertheless, a closer look at the dynamics of the properties of $B_{i}$ may reveal significant quantitative differences.

**Table 1:** Several global properties of the examined innovation networks *A*

	Barabási-Albert	Erdős-Rényi	Watts-Strogatz
Number of agents *N*_*A*_	100	100	100
Number of links between agents *M*_*A*_	200	200	200
Network density *ρ*_*A*_	0.04	0.04	0.04
Average degree *D*	4.0	4.0	4.0
Network diameter $\varnothing $	5.34(5)	7.36(7)	10.6(1)
Average path length *l*	2.952(8)	3.455(5)	5.10(4)
Average clustering coefficient *C*_*l*_	0.160(4)	0.037(1)	0.377(3)
Global clustering coefficient *C*_*g*_	2.32(3)	0.60(2)	4.45(3)
Average betweenness centrality *C*_*B*_	0.01992(8)	0.02506(5)	0.04182(5)
Average harmonic closeness centrality *H*	37.39(8)	33.05(3)	25.0(1)
Degree distribution	Power law	Poisson	Narrow Poisson-like

Uncertainties of the last digits are given in parentheses and represent the standard error of the mean. Values are averaged over 100 simulations with networks set up as described in Appendix D

**Fig. 12:**
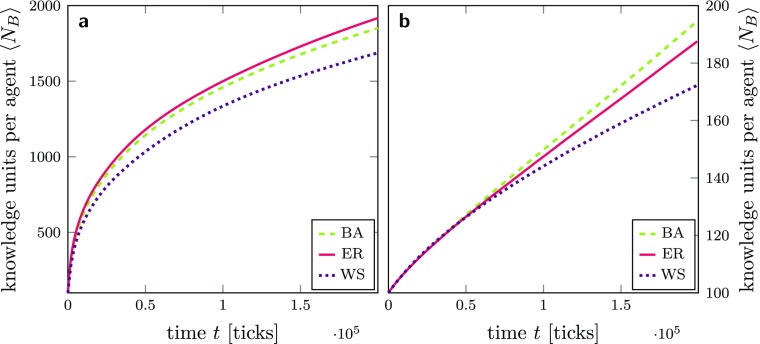
Time series of average knowledge network sizes $\left <N_{B}\right >$ for different innovation network topologies. The different topologies are Barabási-Albert (BA), Erdős-Rényi (ER), and Watts-Strogatz (WS). The lines are averages over 50 simulations with 100 agents (5,000 agents in total) per topology. a: System of 100 agents with a common knowledge background (*p*_*a*_ = 0) and a moderate level of knowledge heterogeneity (*p*_*k*_ = 0.6). b: System of 100 agents with completely different knowledge background (*p*_*a*_ = 1) and a moderate level of knowledge heterogeneity (*p*_*k*_ = 0.6). All agents follow a knowledge exploitation strategy; the compatibility threshold is *γ* = 0.75. The standard error of the mean is smaller than the line width and therefore not shown

In Fig. 12, the left panel (a) shows that in a system where agents share a common knowledge background (*p*_*a*_ = 0), ER innovation networks perform slightly better than BA and WS. The right panel (b) in turn shows that in a system with highly diverse knowledge backgrounds (*p*_*a*_ = 1), BA networks are more efficient than ER and WS. The switch between the diffusion performance of ER and BA in scenarios a and b also relates to the discussion in the literature about the ambiguous effects of “stars” (i.e., agents with a high degree) on knowledge diffusion, depending on them freely giving away their knowledge or trading it (e.g., Bogner et al. 2018; Cowan and Jonard 2007; Müller 2017, Chap. 5; Mueller et al. 2017). Our results are, therefore, also relevant for the discussions about the importance of heterogeneity and diversity of agents within the networks. Hence, with regard to the different performance of the BA network, we can argue that the effect of stars does not just depend on whether they are givers or traders of knowledge but also on the knowledge diversity in the system.

However, comparing these rather artificial network structures may obscure another important element that has received relatively little attention in the literature on innovation networks, namely, that alliances between agents are often formed on the basis of complementary knowledge between partners (e.g., Baum et al. 2010; Cowan and Jonard 2009; Tur and Azagra-Caro 2018). Therefore, in line with the ideas of Joel Baum, Robin Cowan, and Nicolas Jonard (2010), we now additionally compare the diffusion performance in a “compatibility-based” (CB) innovation network that is created with the same number of edges as the other innovation networks based on the assumption that agents must have a certain fit or compatibility in their knowledge to form an alliance (see also Cowan and Jonard 2009).^29^ At this point, we assume that the alliances thus formed remain stable during the simulation for the sake of comparability and, therefore, the CB network also remains static. Yet, we are aware of the fact that cognitive distances between agents may change over time.

**Fig. 13:**
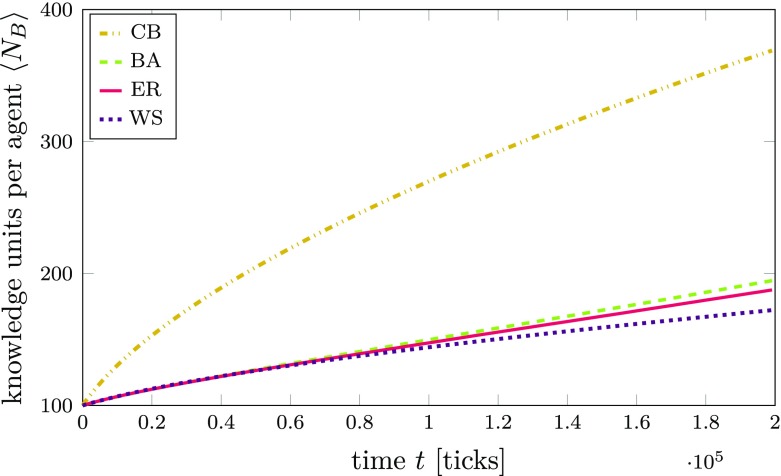
Time series of average knowledge network sizes $\left <N_{B}\right >$ for different innovation network topologies. The different topologies are Barabási-Albert (BA), Erdős-Rényi (ER), Watts-Strogatz (WS), and compatibility-based (CB). The lines are averages over 50 simulations with 100 agents (5,000 agents in total) per topology. Agents are initialized with a highly diverse knowledge background (*p*_*a*_ = 1) and a moderate level of knowledge heterogeneity (*p*_*k*_ = 0.6). All agents follow a knowledge exploitation strategy; the compatibility threshold is *γ* = 0.75. The standard error of the mean is smaller than the line width and therefore not shown

As we can see in Fig. 13, in a scenario where agents have a very different knowledge background, learning in the CB network is much more efficient—and expectedly so—than in the other three network types, as it is much easier for agents to find compatible knowledge among their neighbors. This result is also highly relevant for the overall discussion on the role of efficient network structures, complementary knowledge, and social capital (e.g., as discussed by Baum et al. 2010; see also Cowan et al. 2006, on a related note). Put differently, it may be futile to search for an optimal network structure if the measure of optimal performance exhibits a strong dependence on the properties of individual agents in the network. This finding is not only relevant for our model, where compatibility between agents is implicitly determined by the average compatibility of their knowledge networks. In fact, even in models where knowledge compatibility is not explicitly incorporated, it might still affect the dynamics implicitly. For example, even a simple barter trade mechanism implies some sort of compatibility, since agents have to somehow mutually agree on exchanging knowledge. This, in turn, must be determined by some criterion which is likely to exhibit effects similar to the explicitly incorporated notion of compatibility in our model.

## Conclusion and outlook

With this article, we contribute to the literature on knowledge dynamics in innovation networks. Since knowledge and learning trajectories differ between and within industries, sectors, and regions, models are needed that aid researchers and policy-makers in comprehending these complex processes and their dependence on different initial conditions. Although knowledge is and always has been a somewhat elusive concept, recent advances in network science and (computational) economic modeling have already contributed a lot to better understanding diffusion phenomena. Nevertheless, as elaborated in the introductory and theoretical sections of this article, several important characteristics of knowledge and their implications for modeling diffusion have not been explicitly discussed so far. We have addressed this research gap by developing an agent-based simulation model that captures knowledge diffusion in an innovation network and assimilation depending on compatibility and a shifting focus of attention among networked units of knowledge. This more fine-grained model of compatibility-based, path-dependent learning processes in innovation networks allows us to get a little closer to the qualitative dimension of knowledge representations than some of the other models developed in this field.

So, what have we gained from this added complexity? First, we have been able to analyze the effects of knowledge diversity in different scenarios against the backdrop of agents’ knowledge exploitation versus exploration strategies. Although models are always just a simplification of reality, and there are many other factors to be considered in a real innovation system,^30^ the results of our simulation can already provide us with some relevant insights by pointing toward previously disregarded effects. More precisely, our results relate, among others, to discussions about (cognitive) proximity (e.g., Boschma 2005; Nooteboom et al. 2007) and support the claims that the diffusion of knowledge in innovation networks strongly depends on the diversity of knowledge available in the system. With this model, we are able to distinguish between different levels of knowledge diversity. Most notably, diversity of knowledge *between* agents is also present in previous models of knowledge diffusion; yet, to the best of our knowledge, in previous models the heterogeneity of KUs *within* agents does not influence which part of their knowledge base is more likely to be selected for transmission and, in turn, assimilated. As a consequence of this added layer of complexity, we are now able to see that the advantageousness of knowledge exploitation vs. exploration strategies in terms of knowledge diffusion performance differs considerably depending on the *level* of knowledge diversity. We have observed that in a scenario where agents share a common knowledge background (e.g., because they are rooted within the same technological field), an exploitation strategy is always better in the beginning, whereas exploration leads to more efficient knowledge diffusion in the medium and long run. When agents do not share a common knowledge background, however, the effects are less clear-cut: For situations where the technological fields present within an innovation network exhibit moderate knowledge heterogeneities and where the individual knowledge backgrounds (e.g., technological fields) of the actors differ considerably, exploration is always conducive to their knowledge network growth, whereas for less diverse knowledge backgrounds, knowledge exploitation is more efficient, at least in the short and medium term. Additionally, with our model, we have been able to measure knowledge diffusion differently by focusing on structural effects in knowledge networks (e.g., captured by average degree, weighted density, and modularity of knowledge networks). This is another step beyond the merely cardinal, cumulative measures of many previous models in terms of higher or lower knowledge levels in given categories.

Finally, after comparing knowledge diffusion performance depending on different innovation network topologies, we can see that our results have implications for both researchers and policy-makers interested in the knowledge diffusion performance of an innovation network: If the aim is to find “efficient” network structures to improve collective learning, it may—in some scenarios (e.g., in the case of knowledge exploitation by all agents)—be more important to connect agents with more compatible knowledge (i.e., lower cognitive distance) instead of just focusing on the structural (network) characteristics of the population of agents. Consequently, discussions about an “optimal” network structure should likewise revolve around cognitive distance and knowledge diversity, which ideally also includes diverse cultural knowledge (or a cultural background) that may be more or less compatible to particular areas of economically relevant knowledge. Based on the results of our simulation, we can therefore conclude that knowledge diffusion performance is not only affected by the social structure (or network topology) of an innovation system, but also depends on the distribution of agents with respect to their individual properties (on that topology).

However, we also have to keep in mind that, for our analysis of innovation networks that are formed on the basis of a knowledge fit between agents (i.e., a compatibility-based network topology), we have only considered static innovation networks and different degrees of *initial* cognitive distance. Although we have been able to show that the diffusion performance differs considerably compared to other network topologies, the cognitive distances between agents change over time and, with this, the “optimal” innovation network structure should also change. Consequently, we could argue that if agents’ properties are not static, neither should innovation networks be treated as such. In other words, an “optimal” network structure is very unlikely to be static. In this regard, our model can serve as a suitable starting point to investigate these issues in more detail in future research endeavors.

Although this model already yields pertinent insights, further work is not only possible but necessary to get closer to a complete picture of knowledge dynamics in innovation networks. Future research opportunities include additional analyses with the present model and extensions of the model itself. For example, one potentially insightful additional analysis with the present model could be “heatmaps” for edges in the innovation network in order to capture which communication paths have been used the most and why. This could be particularly interesting in the case where two connected agents have rather incompatible KUs so that we would expect to observe bottlenecks in knowledge diffusion regardless of their formal link.

Potentially informative model extensions include the following examples:

- The next plausible step would be to extend our model to study cases where the topology of the underlying innovation network is not static, i.e., actors can freely choose with whom they form and dissolve alliances. Consequently, in this case, innovation networks would be dynamic and co-evolving with knowledge diffusion and assimilation dynamics at the level of the agents’ knowledge networks. In this context, it would also be interesting to fathom the most promising “strategy mixes” in terms of agents’ knowledge exploitation to exploration ratios.
- Due to the fact that in our model compatibility between KUs is evaluated only between the receiving agents’ currently focused KU with the potentially received one, it would also be attractive to compare the results once we implement the condition that the received KU has to be compatible also with adjacent KUs. In this way, we could capture diminishing marginal benefits of receiving additional KUs.
- Our model should also be extended to allow for differential retention and “forgetting” of knowledge (e.g., deletion of nodes in the knowledge network), for example, in order to analyze the effects of not using certain KUs. Additionally, in future extensions, KUs could also be deleted in favor of “updated” knowledge.
- Another interesting model extension (especially from the standpoint of memetics) may incorporate the creation of new KUs based on variation or recombination of old ones and other “breeding” mechanisms. In this regard, a promising way may include merging our diffusion model with the mechanisms employed in graph-based knowledge creation models (e.g., Morone and Taylor 2010; Vermeulen and Pyka 2017).
- Additionally, the model could be upgraded to allow for and analyze the dynamics of production and consumption of new products (based on the knowledge networks of the agents) in line with previous models (e.g., Mueller et al. 2015; Schlaile et al. 2018).
- As our model uses a simple pull-mechanism for knowledge exchange, it can also be extended to analyze more complex knowledge trade mechanisms (e.g., a barter trade or some kind of payment in return for knowledge) and to compare the results between these mechanisms.
- Innovation networks entail not only knowledge diffusion but also financial flows between agents (e.g., Buchmann and Pyka 2012). Future research should thus be aimed at improving our model by incorporating capital stocks and financial flows as well.
- Another extension that is somewhat related to the previous ones could incorporate competition between agents (e.g., firms) within the innovation network.^31^

## Figures and Tables

**Fig. 14:**
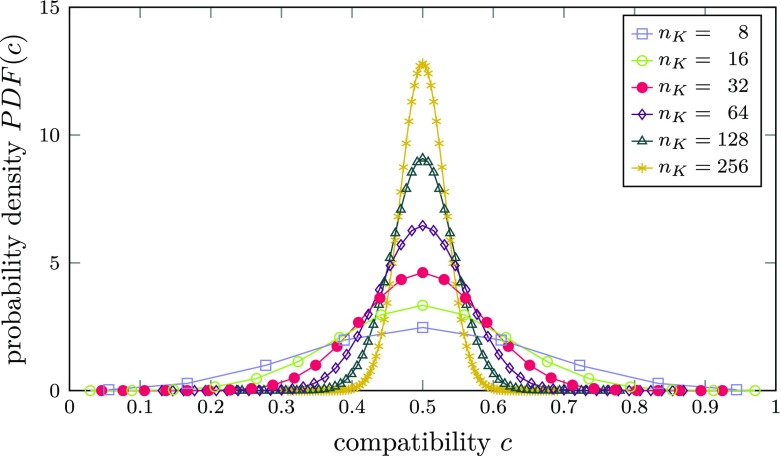
Distribution of compatibilities between pairs of randomly generated KUs for different numbers of bits *n*_*K*_ per KU. The area under the curves is normalized to 1 so that the curves can be interpreted as probability density functions (PDFs). This, in turn, means that the probability *p*_*c*_ for the compatibility of two randomly generated KUs to lie between the threshold *γ* and 1 is $p_{c}(\gamma )={\int }_{\gamma }^{1}\mathit {PDF}(c)dc$. Note that for increasing *n*_*K*_ the resolution of the respective *PDF* increases (depicted by the points on each line), but its shape becomes narrower. Thus, the number *n*_*K*_ should be chosen with care

**Fig. 15:**
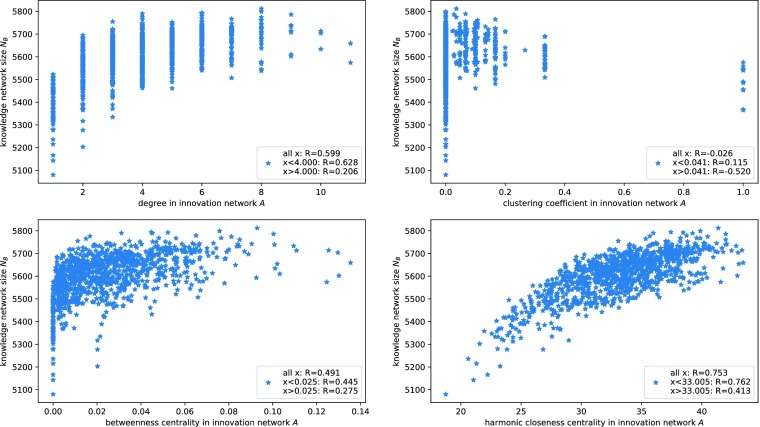
Agents’ number of KUs *N*_*B*_ with respect to their local properties in an innovation network *A* with Erdős-Rényi (random network) topology. The investigated local properties are degree (top left), local clustering coefficient (top right), betweenness centrality (bottom left), and harmonic closeness centrality (bottom right). The numbers *R* reported in the plot legends are Pearson correlation coefficients within the specified range. System parameters are *γ* = 0.75, *p*_*a*_ = 0, *p*_*k*_ = 0.6, knowledge exploitation strategy (random walk on), 10^7^ ticks

## Appendix A: Choice of Model Parameters

The size of the innovation network *A* (i.e., the “macroscopic” network connecting different agents) is chosen as $N_{A}= 100$ in all simulations. This size is small enough to ensure computational feasibility, yet large enough to exhibit the characteristic features of the different network types employed in Section 3.5. However, in order to isolate the effect of network topology on diffusion dynamics to the greatest possible extent, we also fix the number $M_{A}= 200$ of undirected edges connecting the agents. Analogously, the “microscopic” intra-agent knowledge networks $B_{i}$ containing an individual agent’s KUs are initially set up with a size of $N_{B}= 100$ KUs per agent unless stated otherwise.

Our model employs bit strings as a representation of KUs and uses Eq. 1 as a measure for compatibility between any two of such units. However, this definition of compatibility is based on the Hamming distance, which poses problems when it comes to the generation of random KUs: If all KUs are generated randomly from a uniform distribution, the distribution of compatibilities between all pairs of such KUs has the shape of a Gaussian centered at $c = 0.5$. If the integral of the compatibility distribution is normalized to 1, the distribution can be interpreted as a probability density function *PDF*(*c*). This, in turn, means that the probability $p_{c}$ for the compatibility of two randomly generated KUs to lie above the threshold $\gamma $ is given as

5 $$ p_{c}(\gamma)={{\int}_{0}^{1}}P(\gamma)\mathit{PDF}(c)\,dc\overset{(3)}{=}{\int}_{\gamma}^{1}\mathit{PDF}(c)\,dc\,. $$

The width of the *PDF*, however, depends on the number of bits $n_{K}$ per KU (see Fig. 14). For small values of $n_{K}$, the *PDF* is relatively broad. Since bit strings are discrete quantities, the number of different values the compatibility can take on is limited to *n*_*K*_ + 1, though. Thus, the discretization of the compatibility range is rather coarse for small $n_{K}$. Accordingly, the resolution of compatibility improves with increasing $n_{K}$. However, simply increasing $n_{K}$ to a very large number is not advisable, since the Gaussian shape of the *PDF* narrows with increasing $n_{K}$, leaving only a very small range around $c = 0.5$ that actually contributes to the integral. In fact, the *PDF* approaches a Dirac delta function for $n_{K}\to \infty $. This behavior is illustrated in Fig. 14.

A comparison of simulations with different values of $n_{K}$ showed that resolution is only an issue for very small $n_{K}$. Additionally, care must be taken that the maximum number of possible unique KUs $m_{\max }= 2^{n_{K}}$ is much greater than the number of unique KUs in the simulation, which is *m*_sim_ ≤ *N*_*A*_ ⋅ *N*_*B*_. We found that *n*_*K*_ = 32 fulfills these requirements and a further increase of $n_{K}$ leads to no qualitative change in the simulation results when the compatibility threshold $\gamma $ is adjusted accordingly (i.e., it yields the same value of $p_{c}$).

Since we know the initial *PDF* for compatibilities in the system, we can explicitly calculate the probability of existence $p_{c}$ for such edges for any threshold $\gamma $ by evaluating (5). It thus becomes evident that the initial setup of the individual KU networks $B_{i}$ corresponds to a *binomial random graph* (first introduced by Gilbert 1959) constructed according to the $\mathbb {G}_{n,p}$-algorithm (see Frieze and Karoński 2016) with $n=N_{B}$ and $p=p_{c}$, which is equivalent to an Erdős-Rényi random graph. The probabilities $p_{c}$ thus correspond to the expected values of the (unweighted) initial network density $\left <\rho _{B}\right >$.

## Appendix B: Technical framework

Our simulation is written mainly in the C++ programming language^32^ and builds on the software infrastructure provided by the ABM framework repast HPC version 2.1 (Collier and North 2013), yielding a parallelizable and scalable code suitable for large-scale distributed computing platforms. The intra-agent dynamics are modeled using the Boost Graph Library (version 1.60.0) (Siek et al. 2002), and parallelization heavily relies on the Boost MPI and Boost Serialize libraries. All analyses of network properties were performed with self-written tools employing algorithms provided by the Boost Graph Library and a customized version of the Combo code (Sobolevsky et al. 2014). For the visualization of the simulated networks the software Gephi (Bastian et al. 2009) is used. Randomness and reproducability are guaranteed by giving each agent its own instance of a pseudo random number generator (PRNG). The PRNG used here employs the *Mersenne Twister MT19937* algorithm (Matsumoto and Nishimura 1998) provided by the GNU Scientific Library (GSL version 1.16) (Galassi et al. 2009). Reproducability is guaranteed by choosing a random *master seed* at the beginning of the simulation, from which the seeds for all the agents’ private PRNGs are calculated in a deterministic way. The master seed is saved to disk together with all other relevant simulation parameters prior to the start of the simulation so that it is possible to re-run the very same simulation later.

## Appendix C: Dependence of knowledge network size on local properties in the innovation network

In order to quantify the dependence of the number of knowledge units $N_{B}$ on the agents’ location in the innovation network *A*, we calculated Pearson correlation coefficients of $N_{B}$ with respect to the degree, local clustering coefficient, betweenness centrality, and harmonic closeness centrality of the corresponding agents in *A*. The Pearson correlation coefficient *R* assumes a linear relationship between the correlated quantities. Due to the fact that all investigated data sets clearly exhibited non-linear relationships, we did not only compute *R* for the entire data sets, but also separately for the parts of the data where the local properties were below or above the average. Our analyses confirmed the order stated in Section 3.4. Figure 15 shows scatter plots of the number of knowledge units $N_{B}$ with respect to the investigated local properties in *A* for an example system (*γ* = 0.75, $p_{a}= 0$, $p_{k}= 0.6$, knowledge exploitation strategy) after $10^{7}$ ticks. The corresponding Pearson correlation coefficients are given in the plot legends.

## Appendix D: Network-generating algorithms

### Erdős-Rényi networks

First, a set of $N_{A}$ isolated (i.e., unconnected) agents is created. Then, from the candidate set of the possible $M_{\max }$ undirected edges that could potentially connect pairs of agents, we choose one random edge, add it to the network, and remove the corresponding edge from the candidate set. The probability of choosing any particular edge is the same for all edges. Then, with the remaining candidate set of $M_{\max }-1$ edges, we repeat this process until $M_{A}$ edges are added to the network. If the resulting Erdős-Rényi network contains disconnected components, the network is discarded and the whole process is repeated until a network with only one component is found.

### Barabási-Albert networks

A Barabási-Albert network (Barabási and Albert 1999, 2002) is usually created by starting with a connected network of $m_{0}$ agents. Then, individual agents are added to the network successively and connected by $m\leq m_{0}$ nodes until the desired network size is reached, while *m* is fixed for all added agents. The probability of connecting an added agent to any particular pre-existing agent in the network is weighted by the individual degrees of the pre-existing agents. This procedure is also known as *preferential attachment*. Thus, the resulting network cannot have any disconnected components by construction.

Still, we need a deterministic procedure to derive the values of *m* and $m_{0}$ from the given values $N_{A}$ and $M_{A}$. For determining *m*, we introduce the convention

6 $$ m = \left\lfloor\frac{M_{A}}{N_{A}}\right\rfloor\,, $$

where the *floor* operator $\lfloor \cdot \rfloor $ returns the closest integer number smaller than or equal to its operand. In order to be able to derive a simple expression for $m_{0}$, we require the initial network of $m_{0}$ agents to be completely connected, i.e., the number of edges is initially equal to $\frac {m_{0}(m_{0}-1)}{2}$. By additionally requiring $M_{A}=kN_{A}$ with $k\in \mathbb {N}_{+}$, Eq. 6 simplifies to

7 $$ m = \frac{M_{A}}{N_{A}}\,. $$

Since the number of agents successively added to this network is $N_{A}-m_{0}$, we can now derive $m_{0}$ from $N_{A}$ and $M_{A}$:

8 $$ \begin{array}{cll}M_{A} & = \underbrace{\frac{m_{0}(m_{0}-1)}{2}}_{\text{edges of initial network}} + \underbrace{(N_{A}-m_{0})m}_{\text{edges added by preferential attachment}} &\\ & \overset{(7)}{=} \frac{1}{2}{m_{0}^{2}}-\frac{1}{2}m_{0}+M_{A}-\frac{M_{A}}{N_{A}}m_{0} & \qquad | -M_{A}\\ 0 & = {m_{0}^{2}}-m_{0}\left( 2\frac{M_{A}}{N_{A}}+ 1\right) & \qquad | \times\frac{1}{m_{0}}\\ & = m_{0}-\left( 2\frac{M_{A}}{N_{A}}+ 1\right)\\ \Rightarrow m_{0} & = 2\frac{M_{A}}{N_{A}}+ 1 \end{array} $$

### Watts-Strogatz networks

The construction of a Watts-Strogatz network (Watts and Strogatz 1998) is started by generating a regular ring network with $N_{A}$ agents where each agent is connected to its *k* next neighbors (in one direction of the ring) with $k=\frac {M_{A}}{N_{A}}\in \mathbb {N}_{+}$. Then, the edges of each agent in the ring are rewired with probability $p_{\text {WS}}$ to another randomly chosen agent in the network. In order to achieve small-world properties, the rewiring probability is chosen as $p_{\text {WS}}= 0.1$. As in the case of an Erdős-Rényi network, the resulting network might have disconnected components. If this is the case, the network is discarded and the aforementioned procedure is repeated until a network with only one single component is found.

### Compatibility-based networks

First, a set of $N_{A}$ isolated (i.e., unconnected) agents is created and each agent is assigned an AKU with bit randomization probability $p_{a}$. Then, a candidate list *E* is created containing all $M_{\max }=N_{A}\,(N_{A}-1)$ possible edges with weights assigned according to the pairwise compatibility of the agent’s different AKUs. Thereafter, we proceed with the following iterative process with an index *n*, initialized with $n = 0$.

1. Sort *E* by weight in descending order and move the first *n* elements to the end of *E*.
2. Connect agents by the first $M_{A}$ edges in *E*.
3. - The network has disconnected components: Discard the network and shuffle *E* randomly. If the procedure has been repeated a multiple of $N_{A}$ times without success, increase *n* by one. If $n\geq M_{\max }$, throw an error. Otherwise, proceed with step 1.
  - The network is fully connected: Exit procedure.

## Footnotes

1 For more information on the relevance of meso-economic analysis, see Dopfer (2012), Dopfer et al., (2004), or Dopfer and Potts (2008, Chap. 4).

2 For rather general reviews of diffusion literature, we refer the reader to Jackson and Yariv (2011), Lamberson, (2016), and Valente (2006).

3 According to Wesley Cohen and Daniel Levinthal (1990, p. 128), an individual’s or firm’s absorptive capacity is the ability to evaluate and utilize external knowledge, which “is largely a function of the level of prior related knowledge.”

4 For a general review of coevolutionary network dynamics, see Gross and Blasius (2008).

5 For the purpose of our article, we follow Everett Rogers’ notion of an *innovation* as “an idea, practice, or object that is perceived as new by an individual or other unit of adoption” (Rogers 2003, p. 12). One may be tempted to reduce knowledge to the ‘idea part’ of this definition, but—even on a more general note—Dorothy Leonard argues, for instance, that both generation and diffusion of innovation(s) can be viewed “as a process of knowledge generation and transfer” (Leonard 2006, p. 85) and that, regardless of their outward form or medium, innovations can be “considered to be essentially bundles of *knowledge*” (ibid., p. 86, italics in original).

6 However, as John Langrish stresses, it may be (more) suitable to regard memes as “patterns of thought” instead of units (Langrish 2017, p. 315).

7 Note that there are certain reservations (not only) among economists to take up the concept of memes (see, e.g., Hodgson and Knudsen 2010, 2012; Roy 2017), and it can be argued that the model presented below at this stage deliberately neglects some aspects of Darwinian evolution (particularly: extinction and mutation or recombination of these units); so it may not yet be appropriate to call it a genuinely memetic model.

8 Although we do not confine ourselves to individuals as the units of adoption, we focus specifically on the compatibility of a KU with a potential adopter’s prior / existing knowledge, which may also include cultural ideas or “cultural knowledge” held by individuals or organizations (e.g., see Sackmann 1991, on cultural knowledge in organizations).

9 As, for instance, Matthew Crawford explains, “attention ... [can be] treated as a resource—a person has only so much of it” (Crawford 2015, p. 11); and Josef Falkinger (2008) also addresses the problem of “limited attention as a scarce resource in information-rich economies”.

10 As Joel Mokyr declares (in a different but related context), evolutionary systems exhibit “a property of superfecundity ..., that is, there are more entities than can be accommodated, so there must be some selection in the system” (Mokyr 1998, p. 6).

11 Henceforth, for the sake of consistency, we only use the term “knowledge unit” (KU) since the model does not yet capture the whole spectrum of evolutionary dynamics. More specifically, the model does not yet allow for variation and some elements of selection such as forgetting and, hence, differential “fitness” of KUs. Therefore, the term meme may not be fully appropriate at this stage of the model.

12 In the words of Ray Reagans and Bill McEvily (2003, p. 243): “people learn new ideas ... by associating those ideas with what they already know.”

13 Note that alternative conceptualizations of *endogenous* absorptive capacity have also been proposed, for example, by Klaus Wersching (2010) or Ivan Savin and Abiodun Egbetokun (2016).

14 Especially in the context of organizational learning, we may also observe trade-offs between organizational strategies of exploration versus exploitation. We will come back to this in Section 3.

15 See also Bogner et al. (2018) or the discussion of so-called *knowledge communities*, where trust and reciprocity reinforce the openness of knowledge and norms of knowledge sharing (Foray 2004).

16 By way of example, the topology of the innovation network *A* was chosen to be a random Erdős-Rényi graph, and each agent was initialized with 10 KUs generated from a uniform distribution.

17 Consequently, all curves shown in Fig. 3 would collapse onto a single master curve when applying a $\gamma $-dependent scaling factor on the time axis.

18 Due to the fact that for low compatibility thresholds $\gamma $ the resulting knowledge networks are extremely dense, the memory requirements of the simulations become prohibitively large. We therefore had to restrict our investigations to values of $\gamma \geq 0.6$.

19 As, for example, Chris Buskes (2010) explains with reference to Thomas Kuhn’s *The Structure of Scientific Revolutions* (1996), the more specialized the members of a particular scientific community, the more difficult it will be for the paradigms to “interbreed” (not unlike members of different biological species).

20 Because of this, it is no longer possible to collapse the different lines onto a single master curve by means of time rescaling.

21 In the words of Buskes (1998, p. 125): “If the previously trusted corpus of knowledge is left behind, the resulting extended search space may soon provide a new bridgehead for further research in the unfamiliar epistemic setting.”

22 For example, where for *p*_*k*_ = 0.6 a knowledge exploitation (random walk) strategy yielded about 1,000 KUs after roughly 40,000 ticks in the previous scenario, reaching 1,000 KUs in the current scenario requires around five times as long with the same strategy, even if diversity of knowledge backgrounds is relatively low (*p*_*a*_ = 0.4).

23 Even though various measures such as centralities may be applied to describe the “importance” of particular KUs in the knowledge networks by means of their centrality distributions, discussions of meaningful normalization and interpretation would go beyond the scope of the article and should be done in future work.

24 Note that there may be a hypothetical case where two knowledge networks have the same low modularity, one of which has very low edge weights (i.e., consists of rather incompatible KUs) and the other has high edge weights (highly compatible KUs). In this hypothetical case, modularity cannot be meaningfully interpreted as a measure of diversification or specialization taken by itself. However, due to the compatibility threshold $\gamma $ in our model, the extreme cases with low edge weights do not exist in our simulation.

25 One may wonder whether these results may be a mere size effect. However, if we compare the structural properties of knowledge networks of the same size originating from different setups of cognitive distance, we clearly see that this is not the case. For example, for $p_{a}= 0.6$ and $p_{a}= 0.8$ a knowledge network size of a little more than 200 is achieved around time 70,000 and 200,000 respectively. Looking at the according modularities gives us for $p_{a}= 0.6$ at $t=$ 70,000 a modularity *Q* of about 0.6 and for $p_{a}= 0.8$ at $t=$ 200,000 a *Q* of about 0.7.

26 See Appendix C for statistical tests confirming these findings.

27 We are aware that, in reality, innovation networks are often dynamic because alliances between agents may be discontinued due to increasing cognitive overlaps and (re-)established when partners exhibit a sufficient cognitive distance (see also Egbetokun and Savin 2014, their footnote 7; and the discussion in Cowan et al. 2006). However, the static structure of the innovation networks is necessary at this stage to separate structural effects at the level of *A* from structural effects at the knowledge network level (*B*_*i*_). Furthermore, static networks *A* can be interpreted as formal networks that are stable over time due to geographically and contractually binding factors (Bogner et al. 2018; Buchmann and Pyka 2012).

28 Especially in the case of random ER networks, simply generating a random network of $N_{A}$ agents and $M_{A}$ edges poses problems, since the resulting network might contain disconnected components. In that case, one would not simulate a single innovation network of size $N_{A}$, but several separate networks with sizes smaller than $N_{A}$. In order to avoid this problem, we require the networks to consist of one single component. These requirements impose restrictions on the respective generating algorithms, which are discussed in Appendix D. For WS, we used a rewiring probability of $p_{\text {WS}}= 0.1$ for the network to exhibit small-world properties (Watts and Strogatz 1998).

29 For details on how these CB networks are created, see Appendix D.

30 For example, it should be considered whether knowledge can be utilized for new product development, the (search) costs, whether knowledge is tacit or codified, etc.

31 This extension was also suggested by Lorenzo Zirulia (2012) with regard to the model developed by Morone and Taylor (2010).
